# Time-Scaled Evolutionary Analysis of the Transmission and Antibiotic Resistance Dynamics of Staphylococcus aureus Clonal Complex 398

**DOI:** 10.1128/AEM.01777-14

**Published:** 2014-12

**Authors:** M. J. Ward, C. L. Gibbons, P. R. McAdam, B. A. D. van Bunnik, E. K. Girvan, G. F. Edwards, J. R. Fitzgerald, M. E. J. Woolhouse

**Affiliations:** a Centre for Immunity, Infection and Evolution, School of Biological Sciences, University of Edinburgh, Edinburgh, United Kingdom; b The Roslin Institute and Edinburgh Infectious Diseases, Royal (Dick) School of Veterinary Studies, University of Edinburgh, Midlothian, United Kingdom; c Scottish MRSA Reference Laboratory, National Health Service Greater Glasgow and Clyde, Glasgow Royal Infirmary, Glasgow, United Kingdom

## Abstract

Staphylococcus aureus clonal complex 398 (CC398) is associated with disease in humans and livestock, and its origins and transmission have generated considerable interest. We performed a time-scaled phylogenetic analysis of CC398, including sequenced isolates from the United Kingdom (Scotland), along with publicly available genomes. Using state-of-the-art methods for mapping traits onto phylogenies, we quantified transitions between host species to identify sink and source populations for CC398 and employed a novel approach to investigate the gain and loss of antibiotic resistance in CC398 over time. We identified distinct human- and livestock-associated CC398 clades and observed multiple transmissions of CC398 from livestock to humans and between countries, lending quantitative support to previous reports. Of note, we identified a subclade within the livestock-associated clade comprised of isolates from hospital environments and newborn babies, suggesting that livestock-associated CC398 is capable of onward transmission in hospitals. In addition, our analysis revealed significant differences in the dynamics of resistance to methicillin and tetracycline related to contrasting historical patterns of antibiotic usage between the livestock industry and human medicine. We also identified significant differences in patterns of gain and loss of different tetracycline resistance determinants, which we ascribe to epistatic interactions between the resistance genes and/or differences in the modes of inheritance of the resistance determinants.

## INTRODUCTION

The bacterium Staphylococcus aureus can colonize and cause infection in humans, livestock, and other animals. Methicillin-resistant S. aureus (MRSA) presents a significant challenge to public health and veterinary medicine, and of particular concern is the emergence of strains that are resistant to multiple classes of antibiotics. MRSA can be acquired by humans in health care and community settings (1) and is an emerging cause of infection among individuals (and their close contacts) working with livestock (2).

S. aureus clonal complex 398 (CC398), as defined by multilocus sequence typing (MLST) (3), includes MRSA and methicillin-sensitive S. aureus (MSSA) and has been reported in humans and livestock across the globe. While exposure to livestock has been identified as a risk factor for human CC398 infection (2), there have been human cases with no reported livestock contact (4), and the transmission of CC398 between humans has been inferred from epidemiological analyses (5, 6). In humans, invasive CC398 infection has been associated with a range of clinical outcomes, which may be life threatening (7). CC398 may also cause clinical infection in animals and has been associated with bovine mastitis (8).

Studies that have combined MLST sequence data from a number of sequence types (STs) have shown that S. aureus may jump between human and livestock hosts in both directions (9, 10). In an analysis of MLST sequences for almost 700 STs, ST398 clustered with STs that were wholly associated with human hosts (10), and a human ancestral host for ST398 was thus parsimoniously inferred. Microarray studies (6, 11) of CC398 have shown a distinction between livestock-associated infections and those associated with human-to-human transmission in terms of their distribution of mobile genetic elements (MGEs), which has been echoed by studies of the CC398 core genome (12). However, the evolutionary history of CC398 has not yet been studied with quantitative phylogeny-based analyses that explicitly incorporate information about the date, host, and location of sampling.

In contrast to mainland Europe, few publications have described CC398 colonization or infection of humans or animals in the United Kingdom. However, cases of human CC398 infection have been identified by the Scottish MRSA Reference Laboratory (SMRSARL), and CC398 has been reported in two horses in southeast England (13), in bulk tank milk from dairy cattle (14), and in United Kingdom poultry (15). Despite the close association between CC398 and pig farming, in 2008 it was reported that no MRSA isolate, either ST398 or non-ST398, was found on sampled holdings with breeding pigs in the United Kingdom (16). Strains from humans and animals in the United Kingdom have not been included in published genomic analyses of global CC398.

In this study, we performed a time-scaled global molecular epidemiological analysis of whole-genome S. aureus CC398 sequences from humans and livestock, and we discuss the phylogenetic placement of sequenced strains from Scotland. We used quantitative phylogenetic methods to identify sink and source populations for CC398 and formally investigated the evolutionary histories of antibiotic resistance in a phylogenetic framework.

## MATERIALS AND METHODS

### Identification of CC398 isolates from the Scottish S. aureus database.

Thirty-six CC398 isolates were identified from the SMRSARL database, resulting from surveillance between February 1997 and December 2011. SMRSARL receives S. aureus isolates (both MSSA and MRSA) from diagnostic laboratories across Scotland. In addition to isolates from humans, there was also an environmental (cleaning) isolate from a hospital (17) and an isolate from a veterinary laboratory. Isolates were identified as CC398 by searching for CC398-associated MLSTs and *spa* types or, where MLST and *spa* typing had not been carried out, pulsed-field gel electrophoresis (PFGE) profiles and PCR-ribotyping (PCR-R) profiles matching strains already identified as CC398. In the subsequent genomic analysis, membership in CC398 was confirmed by assessing the phylogenetic clustering with sequences from STs belonging to CC398.

The earliest CC398 record in the SMRSARL database was from 2002 and corresponded to an MSSA isolate from an animal in a veterinary laboratory. The earliest Scottish CC398 isolate from a human was an MSSA isolate from a blood sample collected in 2005. The isolates were from approximately equal numbers of men and women, ranging between 0 and 85 years of age. The number of antibiotics to which strains were resistant, as determined by phenotypic testing, ranged from 0 to 8 and included both MRSA and MSSA isolates (see Table S1 in the supplemental material). Seven *spa* types were identified among the sequenced Scottish CC398 isolates (t011, t034, t108, t571, t899, t1255, and t1451), and one of the sequenced isolates had a new *spa* type. There were 6 *spa* types common to both the Scottish CC398 isolates and a previously published global, non-United Kingdom CC398 data set (12).

### S. aureus CC398 DNA extraction and whole-genome sequencing.

Seventeen of the 36 Scottish CC398 isolates were selected for whole-genome sequencing and subsequent molecular epidemiological analysis. Strains were chosen to maximize the diversity in time (isolates from 1998 to 2011) and space (with regard to the Scottish National Health Service [NHS] Health Board) (see Table S1 and Fig. S1 in the supplemental material). The stocked isolates were streaked onto blood agar plates and grown overnight at 37°C. Where strains did not grow in this first instance, an attempt was made to grow them on tryptone soy agar overnight at 37°C. One colony from each plate was selected for DNA extraction. Whole-genome sequencing, using the Illumina Mi-Seq platform, was carried out by ARK-Genomics at the Roslin Institute, Edinburgh, United Kingdom.

### Sequence assembly and alignment.

Short reads from the Illumina sequencing of CC398 isolates from Scotland were aligned against a CC398 reference sequence (GenBank accession number CP003045.1) using the Burrows-Wheeler Aligner (BWA) with the Smith-Waterman algorithm disabled (18), as described in a previous publication (19). The same approach was used to assemble publically available CC398 short reads. A core genome alignment was created from the consensus sequences, with the core genome defined as nucleotide sites shared by all sequences (alignment columns that did not contain a gap character or unknown nucleotide identity). The sequence labeled T7 in a previous publication (12) was excluded due to its conflicting phylogenetic signal.

### Recombination detection.

Previous analysis of CC398 whole-genome sequences (12) identified a region of ∼123,000 bp which had been horizontally acquired from an ST9 donor; as in previous analyses (12, 20), this region of the alignment was not included in the phylogenetic analysis. After removing the ST9 donor region, the alignment of CC398 sequences was further interrogated for recombination using the BratNextGen software (21). Removal of the putative recombinant regions did not have an observable effect on the BEAST maximum clade credibility tree or on the estimates of evolutionary rates and divergence dates and did not reduce the number of variable sites by more than 45 sites (from 5,962). The consistency index for the data set, calculated in PAUP* (22), was 0.9688 for parsimony-informative sites, indicating a low level of homoplasy.

### Preliminary phylogenetic analysis.

Maximum-likelihood phylogenies were constructed using (i) PhyML (23) and (ii) RAxML (Linux version 7.2.8) (24). A general time-reversible nucleotide substitution model was used, with gamma-distributed rate heterogeneity across sites and 1,000 bootstrap replicates. The resulting phylogenetic trees were rooted at the midpoint between the two most divergent taxa in the tree. We investigated the relationship between root-to-tip distances and sampling dates using Path-O-Gen (http://tree.bio.ed.ac.uk/software/pathogen/).

### Time-scaled phylogenetic analysis.

Bayesian phylogenetic analysis was carried out using BEAST (25), with the HKY model of nucleotide substitution and gamma-distributed rate heterogeneity across sites. The uncorrelated lognormal relaxed clock was strongly preferred to the strict clock model under Bayes factor testing. For each combination of priors tested, at least two independent Markov chain Monte Carlo runs of 100 million generations were carried out, with sampling every 10,000 generations. The convergence of the runs was assessed by manually inspecting the chain traces and ensuring that the effective sample size was greater than 200 for all parameters estimated. For all runs, the first 1,000 samples were discarded as burn-in prior to further analysis. Final BEAST runs were carried out using the uncorrelated lognormal clock model with a Bayesian skyline demographic prior.

### Discrete-trait analyses.

An empirical sample of 1,000 trees was generated from the post-burn-in Bayesian skyline BEAST phylogenies. We then used discrete-trait mapping (26) to analyze the dissemination of discrete character states (host species, country of sampling, and presence or absence of antibiotic resistance genes and virulence determinants) across the phylogeny samples. “Markov jumps” analyses (27, 28) were used to count inferred transitions from one state to another (e.g., from livestock to human host or from absence to presence of a particular resistance gene).

### Gene content analysis.

The presence or absence of genes carried on MGEs was investigated by mapping the short-read data for the Scottish and global CC398 isolates to reference sequences for genes of interest: the human immune evasion cluster genes *scn*, *chp*, *sak*, and *sea* (29); *mecA* and *mecC*, which confer methicillin resistance; *tetM* and *tetK*, which confer tetracycline resistance; and concatenated *lukF-PV* and *lukS-PV* for Panton-Valentine leukocidin (PVL). Reference sequences were downloaded from GenBank (http://www.ncbi.nlm.nih.gov/GenBank/) or the KEGG gene database in DBGET (http://www.genome.jp/dbget/), with 100 nucleotides (nt) of flanking sequence at either end (see Table S2 in the supplemental material). We chose the genes to test based on a particular interest in methicillin resistance for S. aureus strains, the historical use of tetracyclines as growth promoters in the livestock industry, the association of PVL with community-associated S. aureus, and previous reports of the human immune evasion cluster genes as markers for human adaptation (29).

Only when the proportion of sites mapped to (excluding the 100-nt flanking regions) and the proportion of identity with the sites mapped to were both greater than 0.9 was a gene classified as “present.” Genes were classified as “absent” otherwise. Similar thresholds have been used in previous analyses of gene content for S. aureus (12), and the concordance with previously published results for CC398 strains was checked where possible. In addition, the results for presence or absence of the genes encoding toxins or antibiotic resistance were compared to the results of laboratory testing (PCR for PVL and phenotypic testing for methicillin and tetracycline resistance) for the Scottish CC398 sequences.

### Nucleotide sequence accession numbers.

The sequencing data for the 17 Scottish S. aureus CC398 genomes have been deposited in the European Nucleotide Archive (project accession no. PRJEB7209; sample accession numbers ERS541584, ERS541585, ERS541586, ERS541587, ERS541588, ERS541589, ERS541590, ERS541591, ERS541592, ERS541593, ERS541594, ERS541595, ERS541596, ERS541597, ERS541598, ERS541599, and ERS541600).

## RESULTS

### Distinct human- and livestock-associated CC398 clades.

We analyzed 17 S. aureus CC398 genomes from Scotland (see Table S1 in the supplemental material), along with 87 previously published CC398 genomes from humans and livestock around the world, spanning a period of 19 years (see Fig. S1 and S2 in the supplemental material), and constructed a maximum-likelihood tree from the shared (“core”) part of the genome (see Fig. S3 in the supplemental material). After rooting on the midpoint, the taxa clustered into two major clades, each with 100% bootstrap support. One clade largely consisted of human sequences (28 out of 29 members of the clade were from human hosts or environments), and the other was mainly associated with sequences of livestock origin (61 out of 76 members of the clade were from livestock hosts or environments). Scottish CC398 sequences from humans fell within both the livestock- and human-associated clades. The isolate from a Scottish veterinary laboratory fell within the livestock-associated clade. The Scottish CC398 sequences were distributed across the maximum-likelihood phylogeny (see Fig. S3 in the supplemental material), suggesting that there has been more than one introduction of CC398 into Scotland. An overall pattern of increasing root-to-tip distance with sampling date was observed (see Fig. S4 in the supplemental material).

### Time scale of the emergence of human- and livestock-associated CC398 clades.

**FIG 1:**
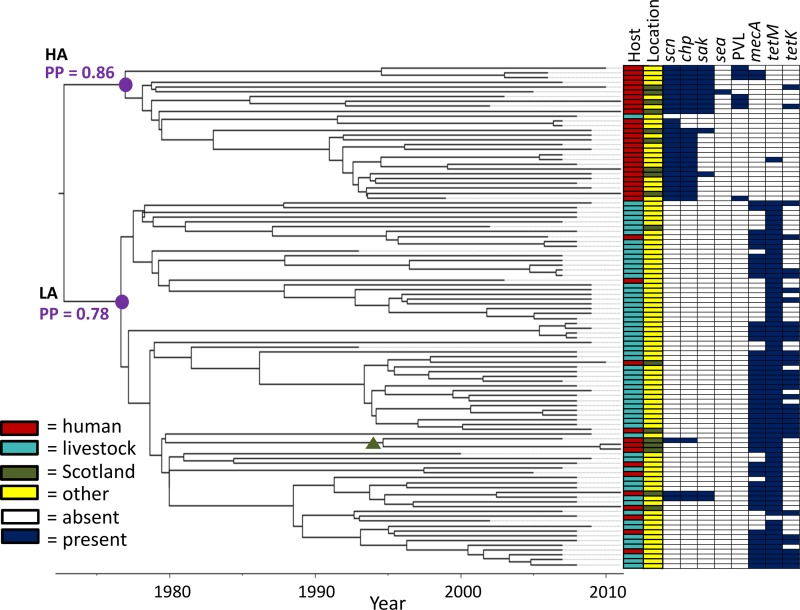
CC398 core genome BEAST phylogeny and distributions of hosts, locations, and genes. The time-scaled Bayesian summary phylogeny for core genome sequences revealed a split into two major clades associated with the host (human or livestock) from which the sequence was sampled. The roots of the major clades are denoted by purple dots, and posterior probability (PP) values are in purple. HA, human-associated clade; LA, livestock-associated clade. The split was also reflected in the presence or absence of genes carried on mobile genetic elements, including known markers for human adaptation (e.g., *scn* and *chp*) (34) and determinants of resistance to methicillin (*mecA*) and tetracycline (*tetM* and *tetK*). Scottish sequences from humans fell in both the human- and livestock-associated clades. A subclade (posterior probability = 1) of three Scottish human sequences within the livestock-associated clade is denoted by a green triangle.

Clustering of CC398 sequences into two major clades—one largely human associated and the other mainly livestock associated—was supported by the BEAST runs using a Bayesian skyline tree prior (Fig. 1). The human-associated clade had a posterior probability (PP) of 0.86, and the livestock-associated clade had a posterior probability of 0.78 (Fig. 1; see Fig. S5 in the supplemental material), indicating support for the separate human- and livestock-associated clades, while reflecting uncertainty in the positioning of certain sequences.

The nucleotide substitution rate was estimated at 1.68 × 10^−6^ substitutions per site per year in the BEAST analysis (95% highest posterior density [HPD] interval = [6.95 × 10^−7^, 2.49 × 10^−6^]). This is broadly consistent with the 95% HPD interval obtained for other S. aureus clones (19). The most recent common ancestor (MRCA) of the CC398 phylogeny was estimated as 1966 (95% HPD interval = [1936, 1985]), and the MRCAs of the human- and livestock-associated clades were estimated as 1971 (95% HPD interval = [1945, 1987]) and 1971 (95% HPD interval = [1945, 1988]), respectively. Thus, although the human- and livestock-associated clades are estimated to have emerged around the same time, there was some uncertainty about the dates of their origin, linked to uncertainty in the rate of molecular evolution.

### Frequent livestock-to-human transmissions of CC398.

**FIG 2:**
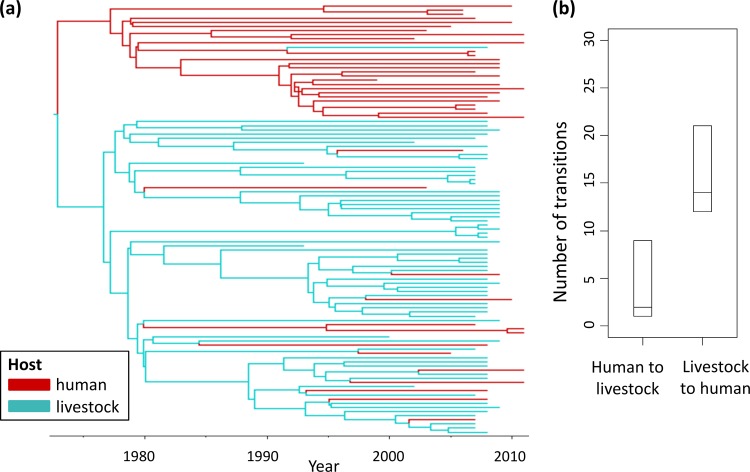
CC398 core genome BEAST phylogeny with discrete-trait mapping by host. (a) The tips of the phylogeny samples were labeled with the host (human or livestock) from which the sequence was sampled and subjected to a discrete-trait mapping analysis to infer ancestral host states. The branches are colored according to the most probable inferred ancestral host state. (b) 95% HPD intervals (upper and lower limits and median, shown on the plot as horizontal lines) were calculated for the number of transitions (Markov jumps) from humans to livestock, and livestock to humans, across the phylogeny samples.

Across the evolutionary history of CC398, there were more inferred transitions from livestock hosts to humans than from humans to livestock (median = 14, 95% HPD = [12, 21] for livestock to humans; median = 2, 95% HPD = [1, 9] for humans to livestock) (Fig. 2). The 95% HPD intervals for the number of transitions between host species do not overlap, indicating that these differences are significant and implicating livestock as an important source of human CC398 infection. The root of the human-associated clade was inferred to have a human ancestral host with a high posterior probability (PP = 0.89), and the root of the livestock-associated clade was inferred to have a livestock host with a high posterior probability (PP = 1.00). There was greater uncertainty about the host state at the root of the entire tree (livestock root PP = 0.72; human root PP = 0.28), and the relatively high posterior probability assigned to a livestock root could have been overly influenced by the oldest sequences in the data set being from livestock.

### Multiple introductions of CC398 into Scotland.

The BEAST phylogeography analysis (mapping discrete country information onto the posterior phylogeny samples) indicated frequent intercountry spread of CC398 in both the human- and livestock-associated clades. Sequences from 19 countries were included in our analysis, which would require a minimum of 18 ancestral transitions between countries across the phylogeny if the sequences clustered perfectly according to country (or 36 transitions if there was complete clustering by country within each of the human- and animal-associated clades). However, on average, 79 (95% HPD = [52, 138]) transitions between countries were required to explain the distribution of locations at the tips of the phylogeny. CC398 sequences from North America, South America, and Europe were found in both the human- and livestock-associated clades, and there was evidence of clustering of sequences from Europe within the livestock-associated clade (see Fig. S6 in the supplemental material).

Introductions of CC398 into Scotland were estimated to have taken place at least 13 times (95% HPD interval = [13, 30]; median = 19) over the evolutionary history of CC398. There was a smaller number of inferred transitions from Scotland to other countries (median = 6; 95% HPD interval = [0, 20]), although there was some overlap between the 95% HPD intervals for the number of jumps in and out of Scotland. The finding that the 95% HPD interval for the number of jumps into Scotland does not include zero supports the hypothesis of multiple introductions of CC398 into Scotland.

### Historical differences in the gain and loss of resistance to methicillin and tetracycline.

Fifty-three percent of the CC398 sequences (7% of the human-associated clade and 70% of the livestock-associated clade) possessed the *mecA* gene for methicillin resistance (Fig. 1) based on our *in silico* testing. The homologue *mecC* was not found among any of the CC398 isolates. Seventy-two percent of the CC398 sequences (4% of the human-associated clade and 99% of the livestock-associated clade) possessed the *tetM* gene for methicillin resistance; 43% of the *tetM*-positive isolates also carried the *tetK* tetracycline resistance gene, and two members of the human-associated clade were positive for *tetK* while being negative for *tetM*. Members of the human-associated clade were significantly more likely to have at least two immune evasion cluster genes (*scn*, *chp*, *sak*, and *sea*) associated with the prophage ϕSA3 (previously shown to be markers for human adaptation [29]) than members of the livestock-associated clade and were more likely to carry the genes for PVL, less likely to be resistant to methicillin, and less likely to carry a tetracycline resistance gene than members of the livestock-associated clade (all tested using Fisher's exact test; *P* < 0.0001).

**FIG 3:**
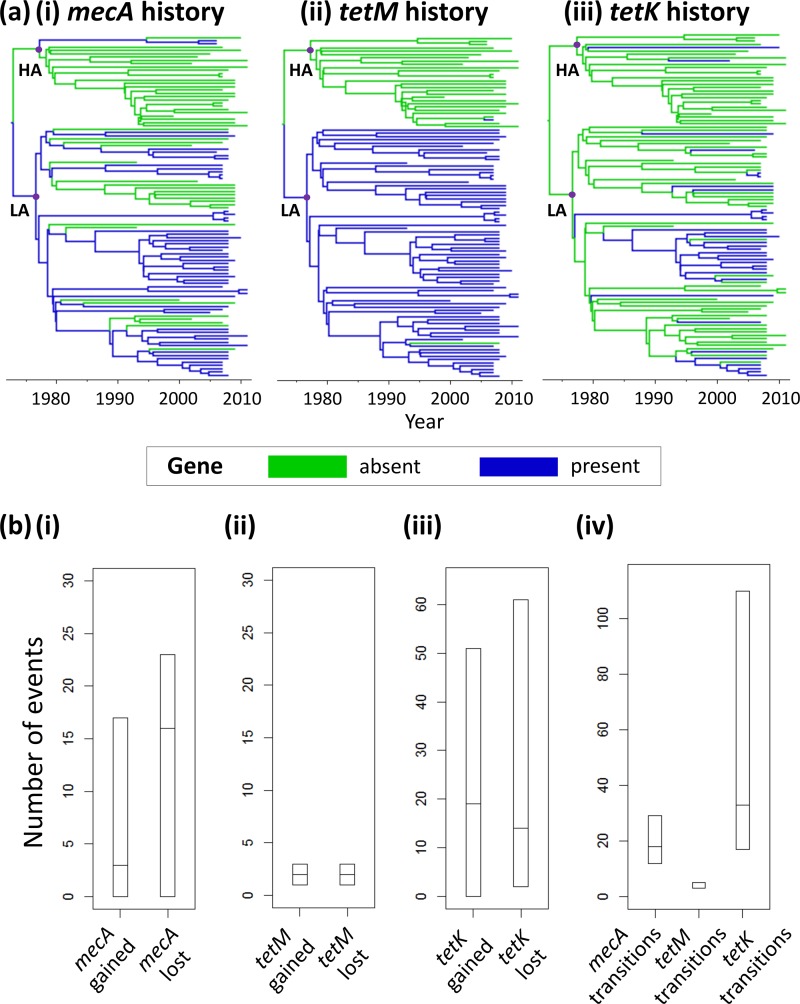
CC398 antibiotic resistance histories for methicillin and tetracycline. (a) The tips of the CC398 phylogenies were labeled with presence or absence of (i) *mecA*, (ii) *tetM*, and (iii) *tetK*. The branches are colored according to the most probable inferred ancestral state (gene present or absent). HA, human-associated clade; LA, livestock-associated clade. (b) Medians and 95% HPD intervals across the phylogeny samples were plotted for the number of gains and losses of (i) *mecA*, (ii) *tetM*, and (iii) *tetK* and (iv) the total number of transitions (gains plus losses) for *mecA*, *tetM*, and *tetK*.

The gain and loss of genes conferring resistance to the antibiotics methicillin and tetracycline was investigated over the evolutionary history of CC398 by mapping the presence or absence of known resistance determinants as discrete traits (Table 1 and Fig. 3). Gain or loss of the *tetM* tetracycline resistance determinant occurred significantly less frequently than gain or loss of *tetK* (median number of *tetM* gain or loss events = 3, 95% HPD interval = [3, 5]; median number of *tetK* gain or loss events = 33, 95% HPD interval = [17, 110]). The median total number of gain or loss events for the methicillin resistance determinant *mecA* was 18 (95% HPD interval = [12, 29]). Almost all (94%) of the *tetK*-positive isolates were also positive for *tetM*. The overall predicted phenotypic history of tetracycline resistance was significantly more stable than for methicillin, as *tetM* was gained and lost much less frequently than *mecA*.

**TABLE 1:** Antibiotic resistance dynamics of CC398

Parameter^*a*^	Value		
	Median	95% HPD limit	
		Lower	Upper
*mecA*			
Total transitions	18	12	29
Gained	3	0	17
Lost	16	0	23
*tetM*			
Total transitions	3	3	5
Gained	2	1	3
Lost	2	1	3
*tetK*			
Total transitions	33	17	110
Gained	19	0	51
Lost	14	2	61

a The number of transitions from presence to absence (loss) or from absence to presence (gain) of genes conferring antibiotic resistance (*mecA* for methicillin; *tetM* or *tetK* for tetracycline) was evaluated across the BEAST phylogeny samples using a Markov jumps analysis. The total number of transitions (the sum of the number of gains and losses) for each antibiotic resistance determinant was also calculated.

## DISCUSSION

In this study, we investigated the evolutionary history of global CC398 strains in a quantitative, hypothesis-driven framework. We shed new light on the transmission and acquisition of antibiotic resistance in CC398 by (i) including CC398 genomes from the United Kingdom, (ii) creating a phylogeny with an explicit time scale to date emergence events, and (iii) analyzing the dissemination of discrete-trait data on host, country, and resistance and virulence determinants across the phylogenies.

Our analyses of CC398 sequences supported the existence of distinct human- and livestock-associated clades that emerged at similar times, with some interspecies transmission in both directions (discussed below). This finding, based upon the core genome, provides further evidence of the distinction between human and animal CC398 strains, which has previously been demonstrated for the accessory genome by microarray studies (6, 11). In accordance with these publications, we also observed a difference between the distributions of MGEs (encoding methicillin and tetracycline resistance and PVL, as well as immune evasion genes) between the human- and livestock-associated clades.

In contrast to a parsimony study by Price et al. (12), which hypothesized a human origin for CC398, we did not observe the human MSSA strains falling basal to the rest of the phylogeny; instead, they fall within the human-associated clade, with a bifurcation at the root of the tree into human- and livestock-associated clades. An advantage of our BEAST study over parsimony is that it does not rely on the choice of an outgroup sequence, which can distort a phylogeny if the outgroup is too divergent (30). Unlike parsimony, BEAST explicitly incorporates information about the sampling dates of sequences and reflects uncertainty in the phylogenetic reconstruction by considering several thousand phylogeny samples, reporting posterior probability values for clades. The discrete-trait analysis we performed did not provide strong support for choosing between a human and a livestock origin, although evidence for a potential human origin for CC398 remains from a study (10) of host distribution across a number of S. aureus STs.

While past studies of CC398 have not included whole genomes from the United Kingdom, our inclusion of 17 previously unpublished Scottish CC398 genomes is made timely by the recent findings of CC398 in bulk milk tanks in the United Kingdom (14), as well as reports of ST398 infection in poultry in England (15). We provide evidence for multiple introductions of CC398 into Scotland, with separate introductions into the human- and livestock-associated clades. However, the source of the Scottish infections (the country and host or source from which they were transmitted) and the extent to which CC398 is able to persist and colonize within the United Kingdom remain unclear.

Of interest is a clade of three Scottish sequences in the livestock-associated clade, with 100% bootstrap support and a clade posterior probability of 1 in the maximum-likelihood and BEAST analyses, respectively (Fig. 1; see Fig. S3 in the supplemental material). The two most closely related sequences in this clade (0831N043 and 0831N044) share an ancestor around 2009 (95% HPD interval = [2006, 2010]) and were sampled approximately 6 months apart from a neonatal ward in Greater Glasgow and Clyde. The third sequence in the clade is an environmental isolate from a cleaning project in a Glasgow hospital and is more distantly related (estimated MRCA with the neonatal isolates = 1994; 95% HPD interval = [1979, 2004]). It is possible that this finding represents the persistence of the livestock-associated strain of CC398 in hospital settings in the United Kingdom, with onward transmission between humans. Such a scenario should be investigated with more intensive sampling.

We used discrete-trait mapping in BEAST to examine the phylogeography of CC398, which has been highlighted as important in the literature (31). We provided evidence for frequent transfer of CC398 between countries in both the human- and animal-associated clades. However, we did not undertake a detailed analysis of the dissemination of CC398 between individual countries, as it would likely be biased by the effects of different sampling schemes from different countries. In future, better-sampled global data sets could be analyzed with a phylogeographic generalized linear model (32) to determine the most important predictors of CC398 spread (e.g., human air travel, livestock movements, and geographical distance between locations) and to examine whether the main predictors differ between the human- and livestock-associated clades.

Phylogenetic discrete-trait mapping methods provide a state-of-the-art framework for identifying sink and source populations for infection (33–35). We provide quantitative support for previous reports that livestock are an important source of CC398 infection in humans by observing a significant number of livestock-to-human transitions within the livestock-associated clade. Coupled with the evidence for higher levels of antibiotic resistance in the livestock-associated clade, this emphasizes the need for strict biosecurity measures in agricultural settings. Humans do not appear to be a significant source of CC398 infection in livestock, although our analysis indicates that such transmission is possible.

Our analysis of antibiotic resistance dynamics across the evolutionary history of CC398 is a novel application of discrete-trait mapping methods and provides a proof of principle for a powerful new way of elucidating historical antibiotic resistance patterns in a population. For example, it allows us to determine whether resistance has arisen multiple times locally or has been acquired once and then spread widely. We noted profound differences in the histories of presence or absence of the tetracycline resistance determinants *tetM* and *tetK* across the CC398 phylogenies. One potential explanation for this is that the *tetM* and *tetK* genes found in S. aureus are inherited in different ways: *tetM* is chromosomally carried on a transposon, whereas *tetK* is acquired from a plasmid (see reference 36 for details). It is possible that the mode of inheritance of the resistance determinant affects its stability, or the ability for it to be gained and lost, within a population. The *tetM* and *tetK* genes also confer resistance through different mechanisms: *tetK* through efflux (removal of the antibiotic from bacterial cells) and *tetM* through protection of the ribosomes from the action of tetracycline (see reference 36) for a discussion).

The *tetM* gene is believed to confer resistance to all antibiotics in the tetracycline group, whereas S. aureus strains carrying *tetK* alone are typically susceptible to some members of the tetracycline group (e.g., minocycline), and higher levels of tetracycline resistance have been found in strains harboring both *tetM* and *tetK* (36). It is possible that there is a tradeoff between the fitness costs to the bacteria of carrying both resistance determinants and the benefit of enhanced antibiotic resistance. Discrete-trait mapping analyses on more intensively sampled data sets could help to shed light on the selective forces underlying rates of gain and loss of resistance determinants in the future. Furthermore, our *in silico* study could stimulate laboratory experiments to investigate how epistatic interactions, antibiotic usage levels, and the mode of inheritance of antibiotic resistance determinants affect their rates of gain and loss. For example, wet-laboratory studies could test whether *tetK* is lost more readily in strains that already possess *tetM* and how this relates to different levels of tetracycline usage.

The methicillin resistance determinant *mecA*, which was gained and lost repeatedly over the evolutionary history of CC398 (Fig. 3 and Table 1), particularly in the livestock-associated clade, is also carried on an MGE—the staphylococcal cassette chromosome *mec* element. Our analysis of gains and losses of resistance determinants revealed striking differences between the histories of methicillin and tetracycline resistance, with the methicillin resistance history being far more dynamic. Tetracycline resistance conferred by the *tetM* gene appears to have been extremely stable over the history of the livestock-associated CC398 clade, while tetracycline resistance determinants have been almost ubiquitously absent from the human-associated clade. It is possible that such differences have been driven by historical and current differences in tetracycline usage between human and animal populations. Despite reports of a decline in the use of tetracyclines in human clinical settings and a ban on their use as livestock growth promoters in Europe since the 1970s (37), tetracyclines are still routinely added to animal feed in other countries, as well as being administered in therapeutic doses on commercial farms (38).

Mapping discrete traits onto phylogenies in a Bayesian framework will provide a new way to test hypotheses about the sources and spread of bacteria, antibiotic resistance, and virulence determinants using data from natural populations. With more detailed data sets, it would be possible to formally investigate the correlation between resistance dynamics and host species or between patterns of resistance to different antibiotics. The relationship between resistance patterns and antibiotic usage levels over time could also be investigated. Genomic analysis will also allow the dynamics of resistance encoded by different mechanisms (e.g., core genome single-nucleotide polymorphisms [SNP] or different types of MGE) to be compared. Understanding the past dynamics of resistance to different antibiotics using discrete-trait analyses could help to inform future strategies for reducing resistance, such as drug cycling. In addition, the findings from discrete-trait mapping studies could be used to generate hypotheses to test through transmission experiments with animals.

In conclusion, we have performed a time-scaled phylogenetic analysis of CC398 and provided a quantitative understanding of the circulation of CC398 through separate human- and livestock-associated lineages, but with livestock also a significant source of human infection. We have also carried out a quantitative phylogenetic analysis of the loss and gain of antibiotic resistance determinants. However, human and livestock populations are linked in many ways, including agriculture, the food chain, and shared environments (39). In the future, further studies of a range of bacterial species and strains, using a large number of sequences from numerous potential sources of infection, are required to develop a more detailed understanding of the spread of bacteria and antibiotic resistance.

## Supplementary Material

Supplemental material
