# Supplementary material for: Time-Scaled Evolutionary Analysis of the Transmission and Antibiotic Resistance Dynamics of Staphylococcus aureus Clonal Complex 398
Source: Appl Environ Microbiol. 2014 Dec;80(23):7275–82. doi: 10.1128/AEM.01777-14 (PMC4249192; doi:10.1128/AEM.01777-14)
Supplement: Supplemental material [file supp_80_23_7275__index.html]

Supplemental material 

# Time-Scaled Evolutionary Analysis of the Transmission and Antibiotic Resistance Dynamics of Staphylococcus aureus Clonal Complex 398

## Supplemental material

**Files in this Data Supplement:**

- Supplemental file 1 -

  Characteristics of sequenced Scottish CC398 isolates (Table S1), reference sequences (Table S2), distribution of CC398 sequence data by host and year (Fig. S1) and by country (Fig. S2), RAxML phylogeny of CC398 sequences from humans and livestock (Fig. S3), root-to-tip distance plots for RAxML phylogeny of *S. aureus* CC398 sequences (Fig. S4), BEAST maximum clade credibility tree of CC398 sequences from humans and livestock (Fig. S5), and CC398 BEAST maximum clade credibility tree colored by ancestral location (Fig. S6).

  PDF, 891K
