# Supplementary material for: Time-Scaled Evolutionary Analysis of the Transmission and Antibiotic Resistance Dynamics of Staphylococcus aureus Clonal Complex 398
Source: Appl Environ Microbiol. 2014 Dec;80(23):7275–82. doi: 10.1128/AEM.01777-14 (PMC4249192; doi:10.1128/AEM.01777-14)
Supplement: Supplemental material [file AEM.01777-14_zam999105814so1.pdf]

SUPPLEMENTARY MATERIAL

Year	Health Board	Organism	Specimen type	*Spa type*	Antibiogram	Antibiogram length	Case gender	Case age
2005	NHS GG&C	MSSA	Blood	t034	Pn	1	F	44
2009	NHS Grampian	MSSA	Swab	t571	PnEr	2	F	34
2009	NHS Lothian	MSSA	Blood	new	Er	1	M	50
2009	NHS Tayside	MSSA	Swab	t1255	Fully sensitive.	0	F	50
2009	NHS NWTC	MRSA	Swab	t011	PnCxCpTe(Mt)	4	F	72
2010	NHS Fife	MRSA	Swab	t011	PnCxErClCpTeKmSt(MtGnNo)	8	M	74
2010	NHS GG&C	MSSA	Blood	t034	Fully sensitive.	0	M	59
2011	NHS GG&C	MRSA	Umbilicus	t011	PnMtCx	3	F	0
2011	NHS Lothian	MSSA	Swab	t034	Pn	1	F	6
2011	NHS GG&C	MRSA	Swab	t899	PnMtCxClTrTeNo(Rf)	7	M	18
2011	NHS GG&C	MSSA	Blood	t1451	Fully sensitive.	0	M	55
2011	NHS Lanarkshire	MSSA	Blood	t571	PnEr	2	F	85
2002	Vet lab: Grampian	MSSA	Swab	t108	Not available	Not available	Animal	
2007	Research project: Glasgow	MRSA	Swab	t034	PnMtCx	3	Environmental	
2011	NHS GG&C	MRSA	Umbilicus	t011	PnMtCx	3	M	0
2011	NHS Tayside	MSSA	Swab	t571	Pn	1	F	82
2011	NHS Tayside	MRSA	Umbilicus	t899	PnMtCxTrTe(Cp)	5	M	0

Table S1: Characteristics of sequenced Scottish CC398 isolates. Data were obtained from the Scottish MRSA Reference Laboratory (SMRSARL). Antibiotic resistance was determined on the basis of phenotypic testing for the following antibiotics: cefoxitin (Cx), chloramphenicol (Ch), ciprofloxacin (Cp), clindamycin (Cl), erythromycin (Er), fusidic acid (Fd), gentamicin (Gn), kanamycin (Km), linezolid (Lz), methicillin (Mt), mupirocin (Mp), neomycin (No), penicillin (Pn), rifampicin (Rf), streptomycin (St), sulphamethoxazole (Su), teicoplanin (Tc), tetracycline (Te), tobramycin (Tb), trimethoprim (Tr). 'Antibiogram' refers

to the concatenated list of antibiotics to which an isolate was determined to be resistant on the basis of phenotypic testing (brackets denote intermediate levels of resistance). For example, “PnCxCpTe(Mt)” denotes an isolate resistant to penicillin, cefoxitin, ciprofloxacin and tetracycline, with intermediate levels of methicillin resistance; since it is fully resistant to 4 antibiotics it would have an antibiogram length of 4. “Case age” is in units of years, with zero being used to denote an infant of less than one year of age.

**Gene to search for**	**Abbreviation used in text**	**Accession no. for genome or plasmid from which gene sequence was extracted**
Staphylococcal complement inhibitor	*scn*	BX571856.1
Staphylococcal chemotaxis inhibitory protein	*chp*	BX571856.1
Staphylokinase	*sak*	BX571856.1
Staphylococcal enterotoxin A	*sea*	BX571856.1
Panton-Valentine leukocidin toxin (based on concatenated lukF-PV and lukS-PV sequences)	PVL	CP003166.1
Methicillin resistance determinant *mecA* (penicillin binding protein 2A)	*mecA*	CP003166.1
Tetracycline resistance determinant *tetM*	*tetM*	AM990992.1
Tetracycline resistance determinant *tetK*	*tetK*	NC_017334.1

Table S2: Reference sequences used to test *in silico* for presence or absence of particular resistance and virulence genes, as well as genes associated with immune evasion in humans. Sequences were downloaded from GenBank or from the KEGG gene database in DBGET (www.genome.jp/dbget/), including 100 nucleotides of flanking at either end. More details about the human immune evasion cluster genes are available in van Wamel *et al*. (2006) *J. Bacteriol.* **188**(4).

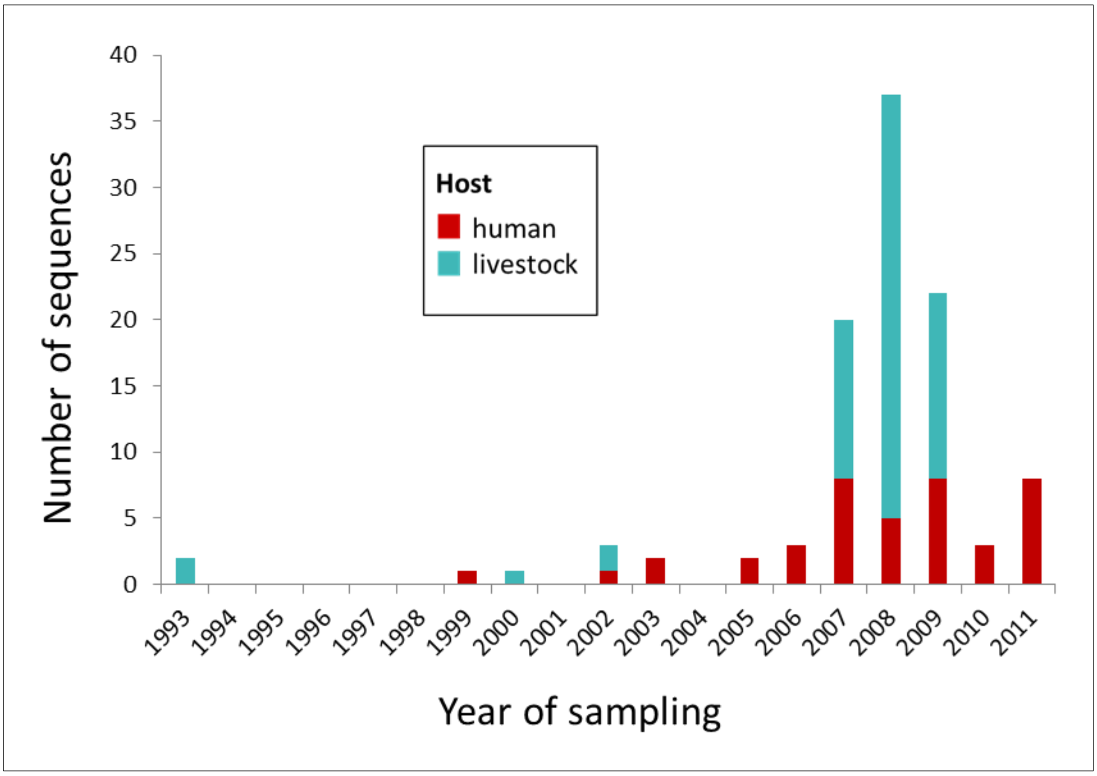

Figure S1: Distribution of CC398 sequence data by host and year. The dataset included previously published CC398 data in addition to 17 newly-sequenced CC398 genomes from Scotland.

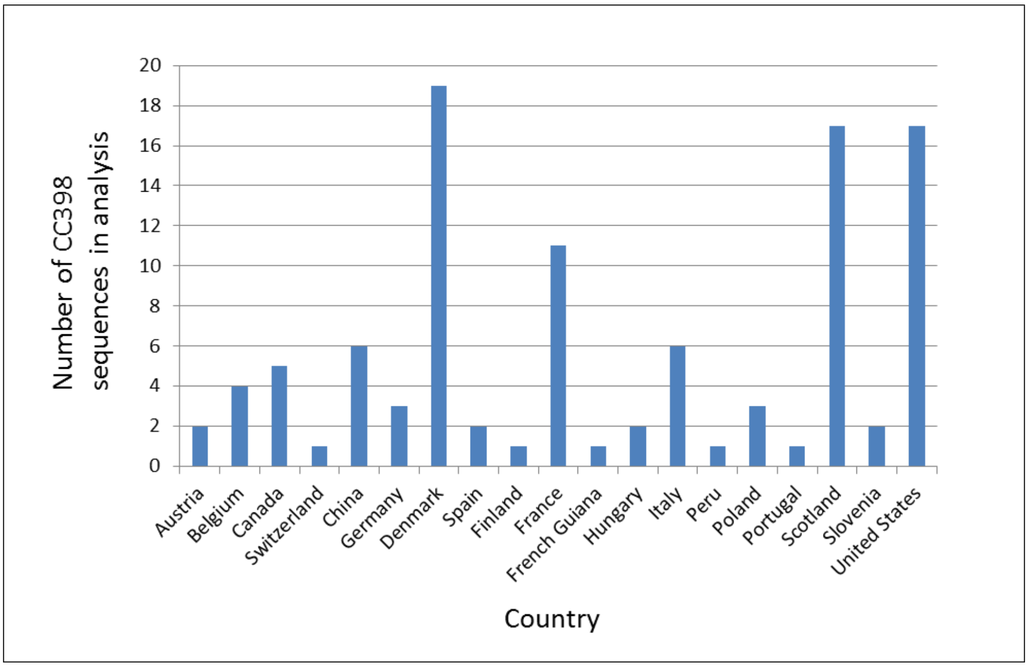

Figure S2: Distribution of CC398 sequence data by country. The dataset included global CC398 genomic data, in addition to 17 newly-sequenced CC398 genomes from Scotland.

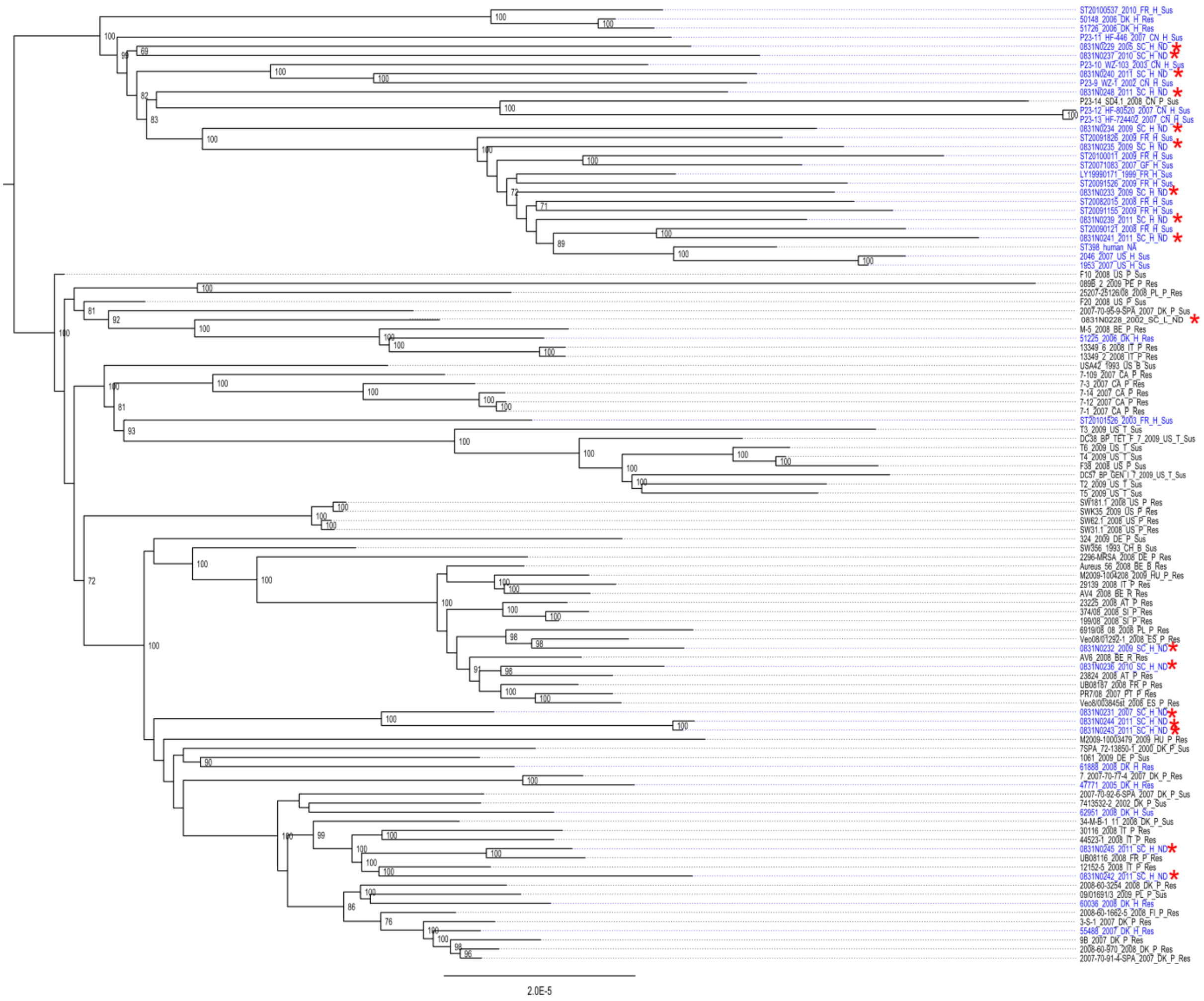

Figure S3: RAxML phylogeny of CC398 sequences from humans and livestock. Bootstrap values of greater than 70% are shown on the tree. Sequences from humans are labelled in blue, with livestock-associated sequences labelled in black. Newly sequenced Scottish CC398 sequences are denoted by red asterisks. The tree has been midpoint rooted.

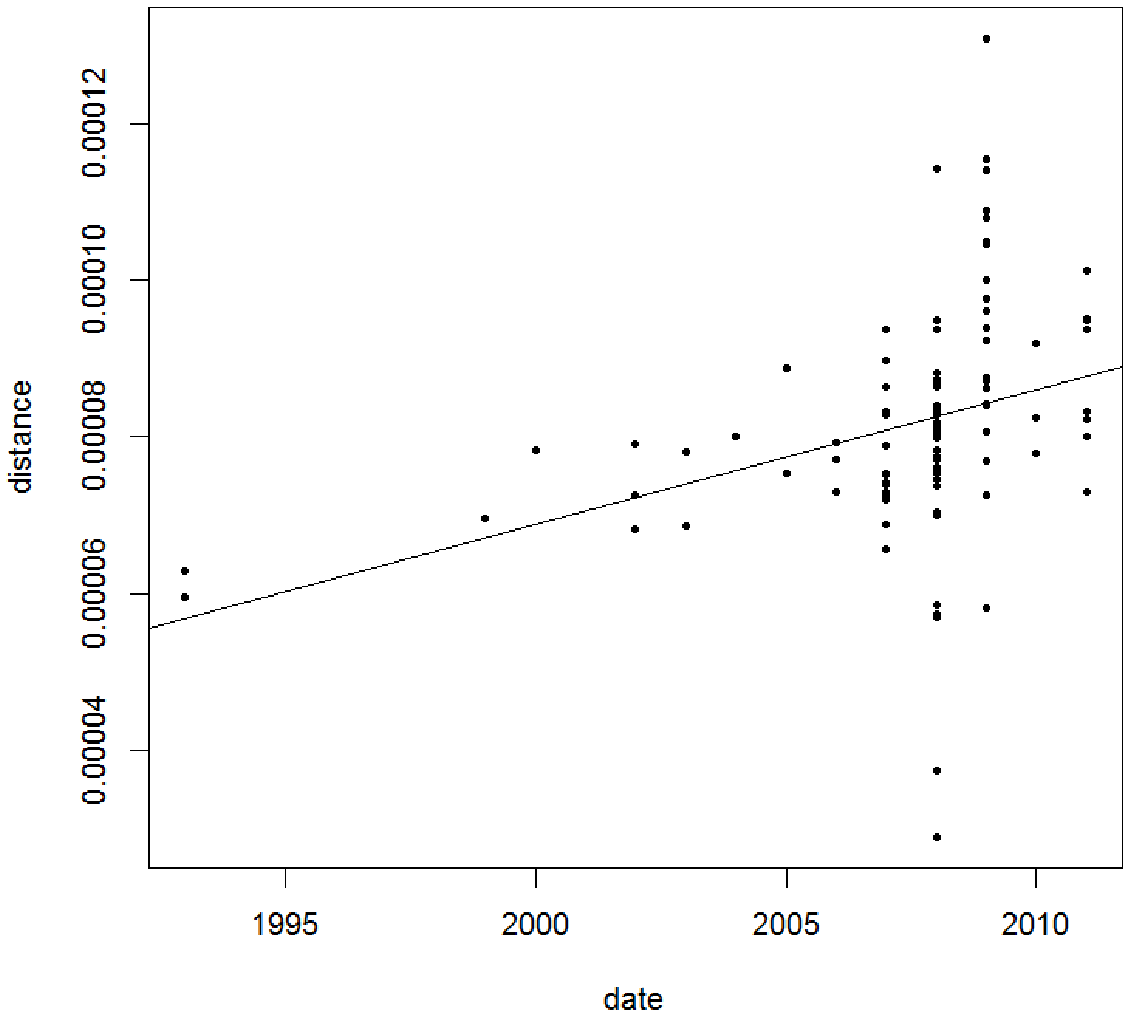

Figure S4: Root-to-tip distance plots for RAxML phylogeny of *S. aureus* CC398 sequences. The distances between the tips and the root of the tree (with the best-fitting root selected) were calculated using Path-O-Gen and plotted against the year of sampling.

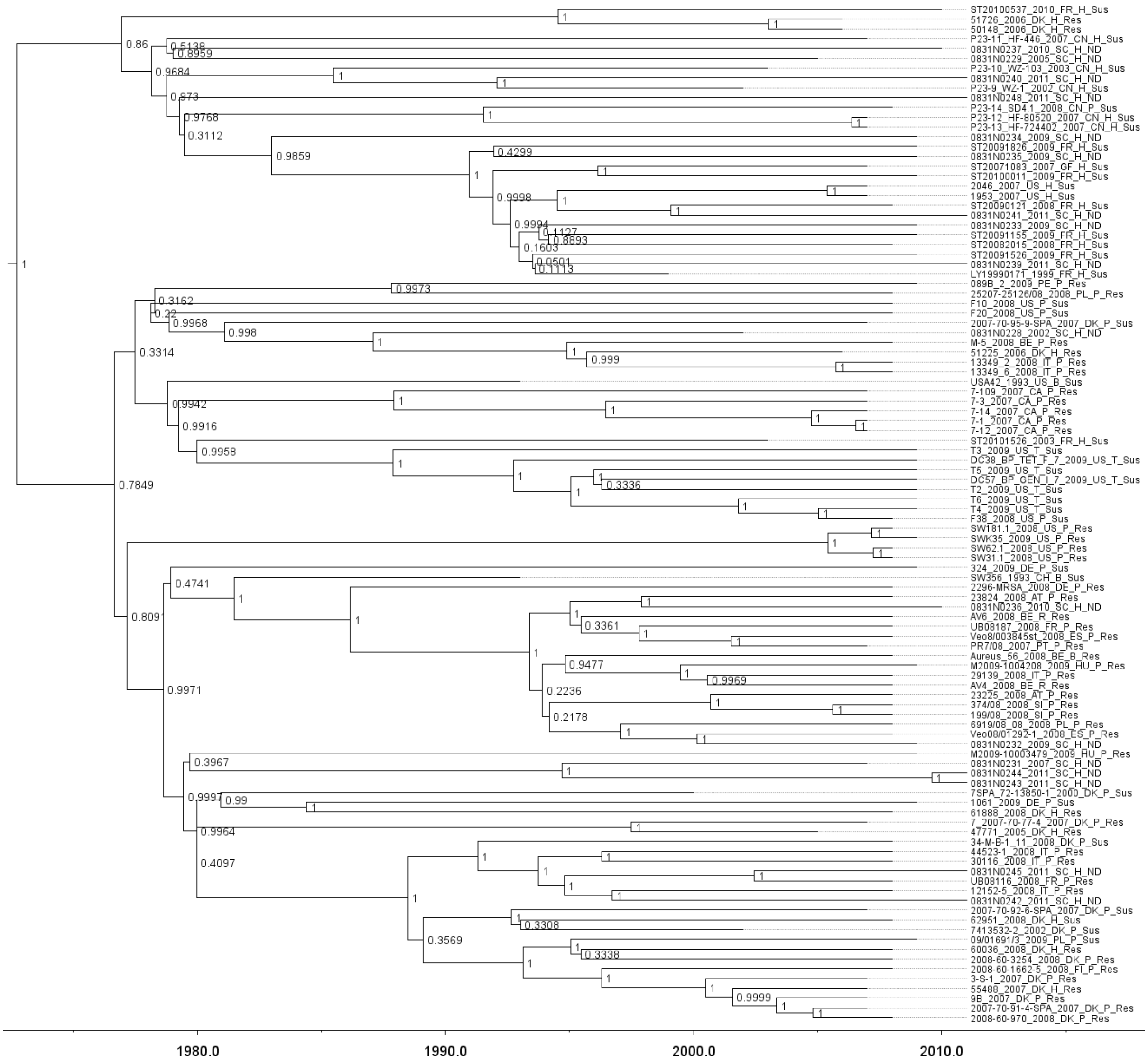

Figure S5: BEAST maximum clade credibility tree of CC398 sequences from humans and livestock. Nodes are labelled with posterior probability values. Newly sequenced Scottish sequences have labels beginning with "0831N0". Other sequences are from Price *et al*. (2012) *mBio* **3**(1) and the first part of the sequence name corresponds to the name in that publication.

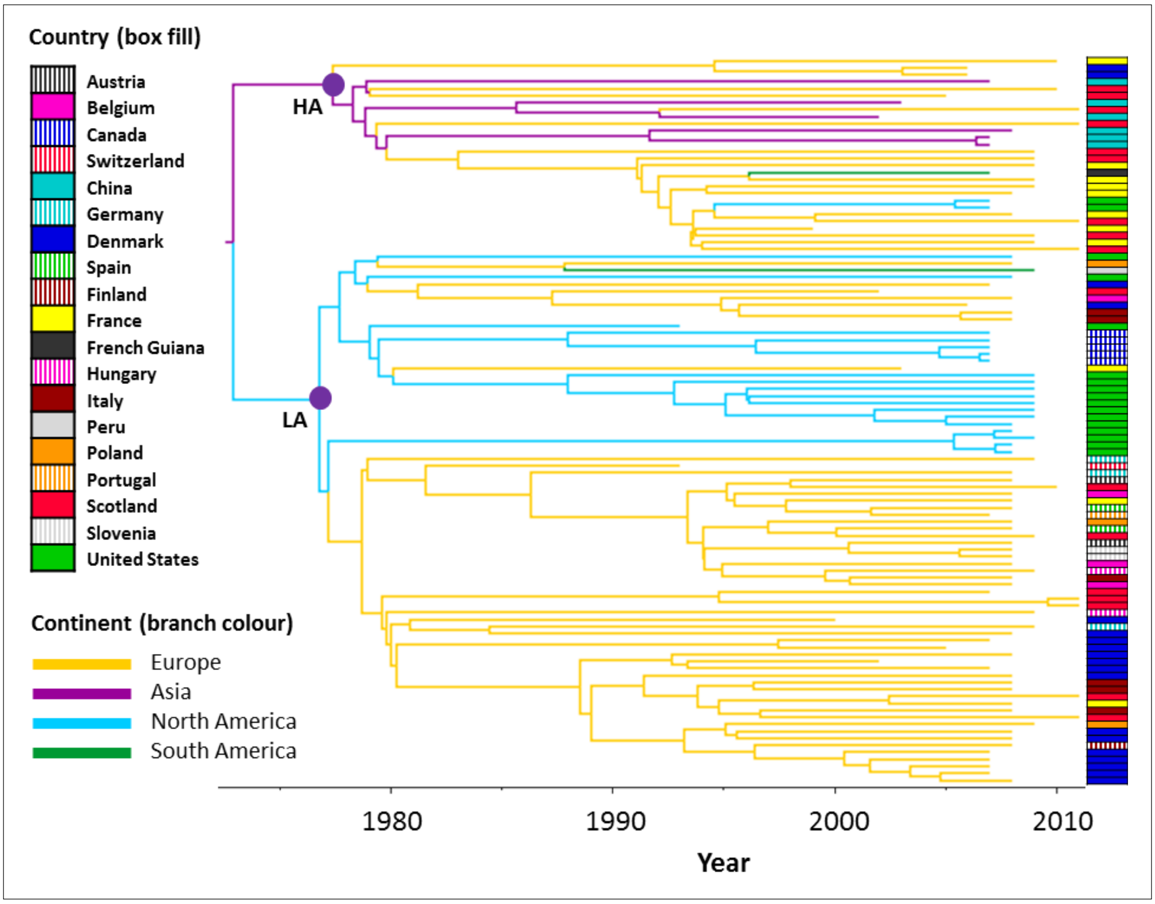

Figure S6: CC398 BEAST maximum clade credibility (MCC) tree, coloured by ancestral location. Branches of the summary phylogeny were coloured according to inferred ancestral location (continent) under an asymmetric continuous time Markov chain model of geographical dissemination. Countries of sampling, corresponding to the tips of the phylogeny, are indicated by coloured boxes at the right hand side of the tree. Country abbreviations: AT (Austria), BE (Belgium), CA (Canada), CH (Switzerland), CN (China), DE (Germany), DK (Denmark), ES (Spain), FI (Finland), FR (France), GF (French Guiana), HU (Hungary), IT (Italy), PE (Peru), PL (Poland), PT (Portugal), SC (Scotland), SI (Slovenia), US (United States). Purple dots indicate the human-associated ("HA") and livestock-associated ("LA") clades.
